# Supplementary material for: Use of Essential Oil Emulsions to Control Escherichia coli O157:H7 in the Postharvest Washing of Lettuce
Source: Foods. 2023 Jun 30;12(13):2571. doi: 10.3390/foods12132571 (PMC10341249; doi:10.3390/foods12132571)
Supplement: Supplementary file 1 [file foods-12-02571-s001.zip › foods-2383034-supplementary.pdf]

Table S1. Log reductions in *E. coli* O157:H7 compared to no-rinse control obtained from surface-inoculated lettuce (romaine, butterhead, and crisphead) immediately after washing treatments (0 h) and after 24 h and 7 days of storage.

**Treatments**	**Storage times**								
	**Romaine (log CFU/g reduction)**			**Butterhead (log CFU/g reduction)**			**Crisphead (log CFU/g reduction)**		
Sanitizers	**0 h**	**24 h**	**7 days**	**0 h**	**24 h**	**7 days**	**0 h**	**24 h**	**7 days**
OR1	-0.92	-1.41	-1.36	-0.15	-0.23	-0.17	-1.45	-1.64	-1.75
OR2	-0.51	-1.97	-1.09	-0.64	-0.80	-1.81	-2.77	-3.03	-2.98
WS1	-0.89	-1.35	-1.49	-0.14	0.11	0.14	-1.63	-2.18	-2.68
WS2	-0.76	-0.81	-0.61	-0.67	-0.43	1.40	-1.93	-1.89	-2.60
OR:WS1	-0.68	-1.36	-1.38	-0.46	-0.27	0.24	-2.51	-3.52	-2.86
OR:WS2	-1.57	-1.05	-1.07	-1.54	-0.85	-1.31	-2.65	-3.41	-2.75
80 ppm PAA	-0.86	-0.62	-1.88	-1.82	-2.09	-2.14	-2.62	-2.55	-2.47
200 ppm Chlorine	-1.61	-1.61	-2.27	-1.62	-1.51	-1.37	-2.70	-2.68	-2.54

Abbreviations: OR: Oregano; WS: Winter Savory; OR:WS: Oregano and Winter Savory mixture; PAA: peroxyacetic acid. EO emulsion concentrations: 1: 0.94 µL/mL; 2: 1.88 µL/mL.

Table S2. Log reductions in *E. coli* O157:H7 compared to no-rinse control obtained from surface non-inoculated lettuce (romaine, butterhead, and crisphead) immediately after washing treatments (0 h) and after 24 h and 7 days of storage.

**Treatments**	**Storage times**								
	**Romaine (log CFU/g reduction)**			**Butterhead (log CFU/g reduction)**			**Crisphead (log CFU/g reduction)**		
Sanitizers	**0 h**	**24 h**	**7 days**	**0 h**	**24 h**	**7 days**	**0 h**	**24 h**	**7 days**
OR1	-4.32	-4.35	-4.27	-4.10	-3.63	-3.28	-3.95	-4.24	-3.98

OR2	-3.82	-3.64	<LD	-4.80	<LD	-5.51	<LD	<LD	<LD
WS1	-2.41	-2.53	-4.03	-1.59	-2.74	-2.83	-3.16	-4.05	-4.15
WS2	-3.14	-4.16	<LD	-4.17	-4.41	-4.46	-3.24	-3.46	-3.51
OR:WS1	-2.73	-3.46	-3.89	-2.55	-3.06	-3.52	-4.73	-4.93	-4.84
OR:WS2	-3.99	<LD	<LD	<LD	<LD	<LD	-5.35	-5.55	<LD
80 ppm PAA	<LD	<LD	<LD	<LD	<LD	<LD	<LD	<LD	<LD
200 ppm Chlorine	-3.01	-3.15	-3.36	-3.83	-1.51	-4.04	<LD	<LD	<LD

Abbreviations: OR: Oregano; WS: Winter Savory; OR:WS: Oregano and Winter Savory mixture; PAA: peroxyacetic acid. EO emulsion concentrations: 1: 0.94 μL/mL; 2: 1.88 μL/mL; LD: Limit of Detection.
